# Trends on Human Norovirus Virus-like Particles (HuNoV-VLPs) and Strategies for the Construction of Infectious Viral Clones toward In Vitro Replication

**DOI:** 10.3390/life13071447

**Published:** 2023-06-26

**Authors:** Emilly Sion, Sharaniza Ab-Rahim, Mudiana Muhamad

**Affiliations:** Department of Biochemistry and Molecular Medicine, Faculty of Medicine, Universiti Teknologi MARA, Selangor Branch, Sungai Buloh Campus, Sungai Buloh 47000, Selangor, Malaysia; emillysion@yahoo.com (E.S.); sharaniza_abrahim@uitm.edu.my (S.A.-R.)

**Keywords:** in vitro translation, infectious viral clones, human norovirus, self-assembly virion, virus-like particles

## Abstract

Most acute gastroenteritis (AGE) outbreaks and sporadic cases in developing countries are attributable to infection by human norovirus (HuNoV), the enteric virus mainly transmitted via fecal-contaminated water. However, it has been challenging to study HuNoV due to the lack of suitable systems to cultivate and replicate the virus, hindering the development of treatments and vaccines. Researchers have been using virus-like particles (VLPs) and infectious viral clones to overcome this challenge as alternatives to fresh virus isolates in various in vitro and ex vivo models. VLPs are multiprotein structures that mimic the wild-type virus but cannot replicate in host cells due to the lack of genetic materials for replication, limiting downstream analysis of the virus life cycle and pathogenesis. The development of in vitro cloning systems has shown promise for HuNoV replication studies. This review discusses the approaches for constructing HuNoV-VLPs and infectious viral clones, the techniques involved, and the challenges faced. It also highlights the relationship between viral genes and their protein products and provides a perspective on technical considerations for producing efficient HuNoV-VLPs and infectious viral clones, which could substitute for native human noroviruses in future studies.

## 1. Introduction

Most acute gastroenteritis (AGE) outbreaks and sporadic cases in developing countries are attributable to human norovirus (HuNoV) infection, the enteric viruses mainly transmitted via fecal-contaminated water. Norovirus infection causes debilitating illnesses, such as nausea, vomiting, stomach pain, and diarrhea, with other symptoms, such as fever, headache, and body aches, following an incubation period between 12 and 48 h [1,2]. Prolonged symptoms usually lead to severe dehydration that requires hospitalization, particularly in vulnerable groups, such as young children, elderly, and people with other diseases [3]. Thus, an outbreak of norovirus imposes significant health and economic burdens; for instance, the infection cases of foodborne noroviruses in the United States were estimated at USD 2 billion in losses due to the lack of productivity and healthcare expenditures [3]. Despite imposing prominent health and economic burdens, neither specific nor effective therapeutics and vaccines are currently available against HuNoV infection.

Studies on the biology and pathogenicity of HuNoV have been limited due to the lack of a suitable cultivation system for norovirus replication, subsequently impeding advances in therapeutics. The problem is also responsible for the lack of a culture-based system for discriminating infectious and noninfectious viral particles. Unfortunately, breakthrough studies on the HuNoV in vitro cultivation systems presented promising results but with slight limitations. Jones et al. reported that the cofactor HGBA type H derived from HBGA-expressing enteric bacteria is required for a successful in vitro replication of HuNoV. However, the cultivation system showed no visible cytopathic effect toward the infection; thus, the detection of the infectious virus was based only upon RT-qPCR analysis. Therefore, the suitability of HuNoV cultivation of the in vitro system tested in their study remains elusive [4]. Another study by Ettayabi and colleagues in 2016 reported a more plausible model but not without challenges, starting from the utilization of enteroids (stem cell-derived organoids), which are costly, and specialized aseptic techniques for the development and maintenance of a viable model. Moreover, the model requires bile acid treatment for the HuNoV GII.3 strain cultivation, which is not necessary for the GII.4 strain [5]. In this regard, the specific bile component responsible for and why the effect was exerted on certain strains remains elusive [5,6]. Indisputably so, without a robust cultivation system, a thorough understanding of human norovirus pathogenicity is limited and might be discriminated by the in vitro environment of the culture system.

Conventionally, a genetic study begins by identifying cells or organisms with phenotype mutation properties and the acquisition of the DNA or protein sequences. This strategy has been applied to investigate many living organisms, including viruses. Numerous studies have reported molecular structure infectivity and interactions of viruses with enveloped or nonenveloped capsid proteins and the host cells [7,8]. Nevertheless, the challenge of obtaining a high concentration of native viruses of interest from clinical samples or the environment hampered studies on certain viruses because virus isolation from clinical samples is time-consuming, expensive, and ineffective for noncultivable viruses, such as HuNoV. The possibility of a successful diagnosis by virus isolation or direct virus detection depends on the attending physician’s attention to sample collection. Ideally, the optimal period to collect samples is at the peak of infection, typically when symptoms initially appear and gradually subside over the following days [9]. Hence, the utilization of infectious cDNA clones [10,11], replicon systems, virus-like particles (VLPs) [12,13], and cultivable animal norovirus surrogate viruses [6] have been employed as alternatives in the study of the virus’s life cycle, and its molecular structures have piqued the interest of researchers [14,15].

This review highlights the approaches to constructing HuNoV infectious particles, strategies for cloning techniques, and challenges involved. The relevance of the viral genome will be discussed concerning the structures, functions, and protein products via in vitro translation techniques with a current perspective on technical consideration toward the production of suitable and viable HuNoV-VLPs. Thus, this review aims to better understand the techniques used in constructing the HuNoV-VLPs as an alternative to fresh virus isolates in the study of norovirus infection.

## 2. Human Norovirus Genome

**Figure 1:**
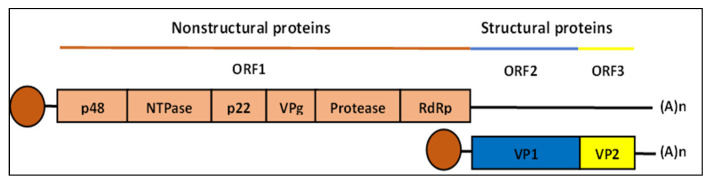
Genome organization of human norovirus (HuNoV). The ORF1 expresses nonstructural proteins starting from 5′ end: p48, NTPase, p22, VPg, protease, and RdRp. The ORF2 encodes for the major structural protein VP1, while ORF3 encodes for the minor structural protein VP2. A subgenomic expressing VP1 and VP2 is linked to VPg as shown below the genomic RNA with the link illustrated as the round shape beside VP1. Adapted from [18].

The HuNoV of the classified genus *Norovirus* belongs to the family *Caliciviridae*, a small, icosahedral, and positive single-strand RNA virus. The RNA genome is about 7.5 kb nucleotides comprising three open reading frames (ORFs), as shown in Figure 1 [16,17]. The ORF1 encodes for nonstructural 6 NS proteins essential for viral replication, including N-terminal protein (p48), nucleoside-triphosphatase (NTPase), 3A-like protein (p22), genome-linked viral protein (VPg), protease (Pro), and the RNA-dependent polymerase (RdRp/Pol) [18]. ORF2 encodes the major capsid protein (VP1) comprising the shell (S), protruding 1 (P1), and (P2) subdomains [17,19], while ORF3 encodes a minor structural protein (VP2) involved in viral RNA encapsidation and stabilization of the VP1 protein [20,21]. Genomic and subgenomic RNAs are covalently linked to a viral protein called VPg at the 5ʹ end and contain a polyadenylated tail at the 3′ end [16].

The genetically diverse noroviruses are classified into 10 genogroups (GI–GX) with 49 genotypes [22]. Nonetheless, only a few genogroups are responsible for acute gastroenteritis in humans, including GI, GII, and GIV, referred to as the human norovirus (HuNoV) [22,23]. The GII.4 genogroup is predominant for viral gastroenteritis outbreaks worldwide [24,25,26].

## 3. Virus-like Particles (VLPs)

The term VLPs has been widely used to describe biological conformations that resemble viral particles distinguishable from various sources, including uncharacterized particles found in biological samples with viral morphologies, viral particles without its replicative system, recombinant viral vector, and structure resulting from self-assembly genes in cloning and in vitro expression systems [27]. VLPs are composed of multiprotein structures forming viral capsid proteins by self-assembly, mimicking the native virion of its origin. These “classical” or noninfectious VLPs are derived from gene-encoding self-assembly-competent proteins without infection and replication capability due to the absence of genetic materials and regulatory proteins [28]. VLP technology has been extensively studied and published for its potential and applications in the biomedical fields, with approximately 467,000 publications in Google Scholar now compared to the 102,000 publications in Google Scholar in 2012 [27].

Theoretically, the formation of VLPs is highly dependent on the protein structures and folding dynamics following a stable expression [29]. It was suggested that Brownian dynamics and interactions between components, including subunits and other viral or nonviral components, drive capsid self-assembly in three phases to ensure minimal free energy in the higher structures. Firstly, there is the nucleation phase, where assembly begins with the formation of a capsid oligomer nucleus from capsid proteins. Secondly, throughout the growth phase, building blocks (protein subunits) of protein monomers or capsid oligomers are introduced to the nucleus. Capsid assembly is completed in the final phase by the insertion of the final building blocks. The assembly process is complex and highly reliant on the structure of the viral protein and the experimental solution environment [29,30,31,32].

## 4. Approaches for the Assembly of HuNoV-VLPs

HuNoV virus-like particles (HuNoV-VLPs), successfully produced in vitro, employed different expression systems to generate the capsid protein. Belonging to the Caliciviridae family, HuNoV has a distinctive feature compared to other animal viruses possessing a single-structure capsid protein, which is common in plant viruses. The capsid protein (VP1) is composed of 90 dimers, is 38.0 nm in diameter, and exhibits a T = 3 icosahedral symmetry, whereby a shell (S) domain and protrusion domains (P1 and P2 subdomains) are connected by a short hinge region [33]. These domains are similar to the tombusvirus capsid structure, e.g., tomato bushy stunt virus and turnip crinkle virus, but with an addition of basic internal region (R domain) involved in RNA binding, yet not found in the norovirus capsid [19].

The S and P domains are two regions that make up the capsid protein when expressed in a suitable expression system [19,34]. It was reported that the reduction in amino acid sequence in the subgenomic structures may or may not disrupt the assembly of protein capsids, which usually depends on the specific location of the missing amino acids. As extensively illustrated in [19], a mutant with 98 amino acid deletions (NT98) in the N-terminal S domain showed an obvious disruption in VLP assembly. In contrast, a mutant with deletions of only 20 (NT20) and 34 (NT34) amino acids did not interrupt the VLP assembly sedimentation, particularly NT20, where the particle yield in terms of morphology obtained was comparable with the morphology of a full-length VLP construct analyzed by electron microscopy.

Mutants with deletions in the C-terminal of the P-domain have also been constructed to illustrate the effects on VLP structure assembly [19]. This construction focused on the P-domain region responsible for the formation of the arch structure of the VLPs. The arch structure is the result of the interaction between subunits of the distal domains (P2) and a central stem domain (P1) in the protruding domain [33]. A deletion in the C-terminal of P1 affects the capsid’s structural stability and morphology, corresponding to the lack of amino acid numbers. Larger particles and less evident arches were obtained when about 20 to 74 amino acids were deleted at the C-terminal. Nevertheless, more deletions of approximately 230 to 303 amino acids in the regions caused the formation of VLPs that lack arch characteristics, resembled by the smoothing and shrinkage of particles observed compared to those in the full-length capsid protein VLPs [19]. This evidence suggests that having a full-length subgenomic sequence of HuNoV is essential to elucidate successful VLP products.

## 5. Factors Influencing In Vitro Assembly

### 5.1. The pH

The optimum acidic or alkaline pH condition for in vitro assembly is crucial for capsid protein formation, whereby some particles can assemble in an acidic or a basic pH. For instance, the capsid proteins of cowpea chlorotic mottle virus (CCMV) were successfully assembled in vitro by acidifying the proteins at approximately pH 5 but sharply lost activity as the pH increased [31]. Similarly, the process of rotavirus VLP self-assembly occurred at an acidic condition near pH 4.5–5.0 [35]. In contrast, the in vitro formation of adeno-associated virus serotype 2 (AAV2) VLPs require a basic condition (pH 9) [36]. The pH of the solution determines the charge type of the proteins, i.e., positive or negative, thus affecting electrostatic interaction [32] during capsid protein in vitro assembly and consequently affecting the protein conformation stability.

The formation of capsid protein can occur in more acidic or alkaline pH, even though the viral particles originate from the same family. Samandoulgou et al. demonstrated that a minimal impact on the secondary structure of VLPs representing human noroviruses (GI.1 and GII.4) and feline calicivirus (FCV) occurred when exposed to an acidic and slightly basic pH [37]. When exposed to variation in pH alone, no significant effect was observed on the secondary structure of Norwalk virus VLPs; however, at a pH of 8, a small loss in α-helices and a corresponding slight increase in the disordered structure were reported [38]. Nonetheless, a dramatic change in the secondary structures of VLPs was reported when exposed to a combination of either temperature with pH or temperature with ionic strength. A higher temperature (72.8 °C) combined with acidic pH caused a major disruption in the ordered structure of the GII.4 particles, but a combination with a pH ranging from 6 to 8 caused a substantial loss in the GI.1 and FCV secondary structures [37].

### 5.2. Ionic Strength

Capsid assembly in vitro is also influenced by the level of ionic interactions in the solution environment. Charge, or the ionic strength, plays a significant role and predominantly influences the polymorph stability of the protein by affecting the electrostatic interaction range and the stabilizing effect of calcium ions binding to the capsid. Salts interact with charges on the protein surface, alter the water shell, reduce hydrophobic exposure, and, as a result, affect protein stability [39]. In the case of HuNoV, the variations of ionic strength resulted in visible changes to the VLP secondary structures, ranging from β-strand to α-helix in GI.1 particles and presented minimal losses in GII.4 particle structure [37]. Increased ionic strength favored helix formation in GI.1, where the condition of the solvent favors intrapeptide hydrogen bonding. However, the increasing ionic strength in GII.4 did not promote helical conformation, which could be attributable to the overall VLP stability [37], yet increased stability was reported at higher concentrations of NaCl [40].

### 5.3. Temperature

A suitable reaction environment temperature also influences capsid assembly, particularly at lower temperatures, as it can prevent protein aggregation and chemical breakdown. For instance, a high yield of in vitro-assembled monkey erythroparvovirus 1 (B19) VLPs were obtained at a low temperature (4 °C) compared to a higher temperature (>37 °C), whereby a higher VLP aggregation was induced and VLP instability occurred [41]. In contrast, the formation of Rous Sarcoma virus capsids in vitro were highly increased at physiological temperatures (34–42 °C); however, the amount of neutral salt in the medium influenced the result of the assembly process [42]. No significant alteration was observed in VLP secondary structures at a lower temperature (4 °C) concerning HuNoV-VLPs (GI.1 and GII.4). However, an apparent shift occurred at higher temperature (>65 °C) treatments. For GII.4 particles, temperatures over 72.8 °C and an acidic pH resulted in the most significant loss of the ordered structure [37].

### 5.4. Nucleic Acids

Essentially, nucleic acids profoundly impact in vitro assembly of certain capsids as capsid assemblies are directly supported by RNA or DNA. For instance, the assembly of a well-studied single-stranded RNA virus model, CCMV VLPs, depended on the optimized strength of interaction between capsid protein (CP) attraction relative to CP–RNA attraction. The capsid assembly will not occur when the interaction is too weak; however, too strong an interaction will cause the assembly to suffer from kinetic traps [43]. Moreover, a sufficiently high mass ratio of protein/RNA is required to completely package the RNA CCMV VLPs [44]. Likewise, other viruses also require nucleic acids to form in vitro VLPs, such as the formation of Gag proteins into human immunodeficiency virus (HIV-1) VLPs in a defined system [45]. Meanwhile, a dsDNA is needed for an efficient simian virus 40 (SV40) VLP assembly [46], and an RNA structure was required for the hepatitis C virus (HCV) nucleocapsid-like particle assembly [47]. Regarding HuNoV-VLPs, a complete subgenomic consisting of a complete VP1 capsid gene is essential for the formation of VLPs [34,48]. Nonetheless, alteration in nucleic acid length prior to assembly should be considered as it may affect the morphology or application of the VLPs [32]. For example, the addition of or reduction in nucleic acids in human norovirus assembly results in various sizes of capsid proteins [19].

## 6. Expression System for HuNoV-VLPs

The success of VLP production is not dependent on having a complete structural gene alone but also on other factors, such as a suitable expression system. Different expression systems have been developed to produce the capsid in the form of VLPs. An expression system is a system used for protein production that comprises the gene encoded by DNA, the molecular machinery required for in vitro transcription, and the translation of the desired gene into protein using the reagents provided, which corresponds to the host cells. Fundamentally, the production of recombinant protein requires three essential components. Firstly, the energy source and machinery for protein production, i.e., a biological environment. The necessary framework for protein synthesis can be from the range of expression host cells or cellular extracts, e.g., cell-free protein expression systems. Secondly, a vector that facilitates the introduction of genetic material into the cell containing regulatory parts that allow replication of the genetic material and selection markers for maintenance. Lastly, the expression cassette is fused into the vector. The genetic material design of the expression cassette is specific to the choice of the expression host system as the necessary components for transcription and translation of the desired protein significantly depend on the gene sequences that control the expression reaction [49,50,51]. Nevertheless, the choice of an expression system depends on protein types, specific applications, and desired yield. Furthermore, modular regulatory elements in expression cassette design play an important role in successfully producing VLPs or recombinant proteins. The different expression systems used in the construction of HuNoV-VLPs are summarized in Table 1.

## 7. Infectious Viral Clones

Understanding the molecular basis of viruses is critical for developing potential viral vaccines. Nonetheless, most viral research limitations are due to the lack of fresh viral isolates, subsequently impeding further studies. Recombinant DNA technology provides a promising alternative through the construction of a viral infection clone.

An infectious viral clone could be synthesized in vitro from a full-length cDNA using reverse genetics technology, also known as in vitro transcription [60]. Infectious clones could infect and subsequently replicate in the host cells, which can also serve as a delivery system for a target gene. The latter refers to vectors that carry the copy of full-length viral genomes with hybrid genes, resulting in propagation-competent chimeric virus progeny, in addition to a vector that carries nonreplication-competent gene fragments but can infect target cells [28]. Moreover, the infectious clones were useful in studies of virus life cycle modeling systems that combine minigenome, transcription, and replication of competent virus-like particle approaches [15,61].

The assembly of the capsid, membrane coat (for the enveloped virus) and the packaging of the nucleic acid (NA) genome into the capsid protein are the required steps in the formation of an infectious virion. Many single-stranded genome viruses can self-assemble around their NA without ATP energy [43], which explains the minimum free energy in the microenvironment, illustrated by [62], where infectious virions assemble spontaneously in a reaction mixture of tobacco mosaic virus RNA and capsid proteins. On the contrary, double-stranded DNA or dsRNA is rigid and has a high charge density, which mostly prevents spontaneous NA encapsidation; thus, it requires more processes for a complete infection virion assembly. In the case of dsDNA, this phenomenon causes pro-capsid construction by viruses, followed by packaging via an ATP-hydrolyzing molecular motor that pumps DNA into the capsid [63].

**Figure 2:**
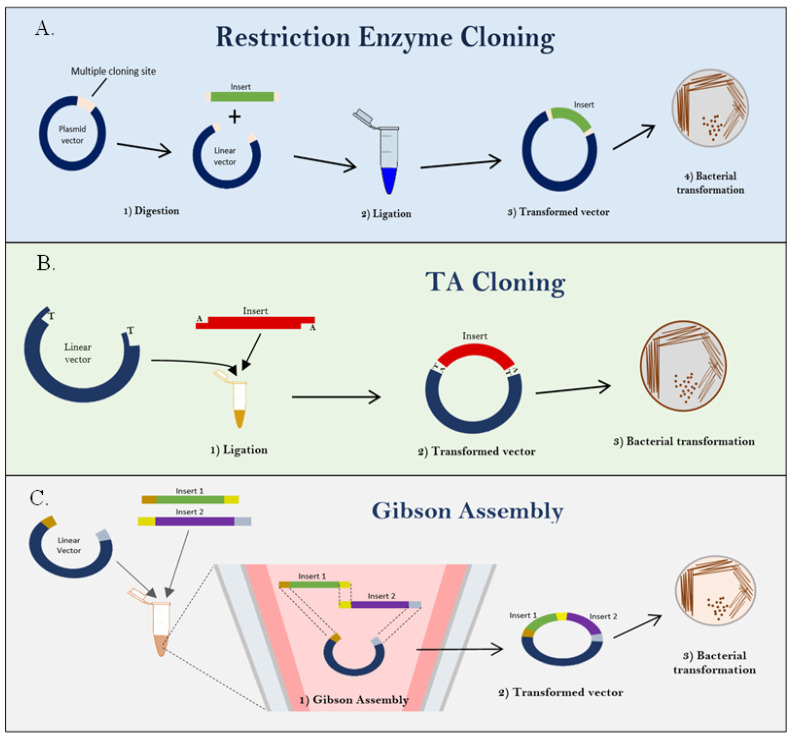
Overview of the common cloning methods. (**A**) The plasmid vector is linearized by digestion with restriction enzyme (RE) on multiple cloning sites producing “sticky ends”. Foreign DNA molecules or inserts are digested with the same RE producing “sticky ends” complimentary to the plasmid vector. The transformed vector is cloned into competent cells for selection of cells with transformed vectors that can grow on the selective media. The illustration was adapted from [65]. (**B**) The DNA insert with 3′ tail consists of a 3′-OH generated by PCR. Blunt-end linearized vector is prepared by ddT tailing with terminal transferase compatible with the insert ends. Ligation of the ddT-tailed vector and DNA insert are due to the formation of phosphodiester bonds between the 5′-P of the vector and the 3′-OH group of the PCR product. Successfully ligated product can be transformed into competent cells. Growth colony on selective media indicates cells carrying the transformed vector. The TA cloning illustration was adapted from [66]. (**C**) Linearized plasmid vector and DNA inserts with respective overlapping regions are assembled in a single isothermal reaction. This method requires a 20–80 bp sequence overlap at the end of the DNA elements to be assembled. The assembled product can be stored in −20 °C or directly transformed into competent bacterial cells. Illustration was adapted from Gibson Assembly in New England Biolabs, n.d. Retrieved 16 December 2021 from https://international.neb.com/applications/cloning-and-synthetic-biology/dna-assembly-and-cloning/gibson-assembly. Copyright 2021 from New England Biolabs.

Conversely, in developing an infectious clone for HuNoV, Oliveira et al. demonstrated that a complete genome of the virus was required with a suitable expression vector comprising efficiently controlled promoters. The seamless cloning system, i.e., the Gibson assembly method (Figure 2C), was adopted to produce the infectious clone virion resembling the native virus without alteration in its genome. In mammalian cells, protein expression is commonly driven by promoter elements, i.e., cytomegaloviral (CMV) promoter, for efficient in vitro transcription [64]. Meanwhile, the self-cleaving ribozyme Hepatitis Delta virus (HDV) was shown to initiate the expression of human norovirus capsid protein [11].

## 8. Approaches for the Construction of Infectious Clones

The most common in vitro methods in the production of recombinant DNA include restriction enzyme cloning, TA cloning, and the most recently discovered Gibson assembly. The restriction enzyme cloning utilizes specific nucleotide sequences, or restriction enzyme sites, to incorporate the gene of interest into a vector using restriction enzyme proteins (Figure 2A). In contrast, TA cloning is one of the simplest and most efficient methods for cloning PCR-generated DNAs with unknown DNA fragment sequences and constructing a DNA library. This system utilizes PCR-amplified products that leave the 3′A tail and fuse it with a linearized cloning vector with prepared 3′T overhangs (Figure 2B). Next, the isothermal assembly method, widely known as the Gibson Assembly method, is a cloning technique that enables the cloning of two or more fragments into vectors without using suitable restriction sites or overhang dependence. The fusion of the insert and vector is generated by overlapping regions, which are later ligated by a specific enzyme in the reaction (Figure 2C). The common cloning techniques used to generate infectious viral clones or VLPs are summarized in Table 2.

## 9. In Vitro Translation System

An in vitro translation system was developed for the mRNA identification and product characterization; thus, it has been used extensively for viral gene identification and validation of cDNA clones. The system was also used to translate mRNA molecules bound with polyribosomes, derived from in vitro transcription or purified from tissues or cell lines [80]. Therefore, in vitro translation allows the synthesis of recombinant proteins without the use of organisms or cells as hosts. Two general systems have been developed: protein synthesis using purified recombinant elements (PUREs) and extract-based systems [81]. In the PURE system, the translation machinery involves translation factors, transfer RNAs (tRNAs), components for mRNA template formation, and ribosomes purified from cells separately and mixed in vitro to create a translation-competent environment [82]. Meanwhile, the extract-based system involves lysis and the extraction of the raw intracellular environment, supplementation with energy-generating components, such as ATP, GTP, creatine phosphate, creatine kinase, and protein synthesis from a DNA template [81,83].

Notable considerations are essential for obtaining an efficient in vitro translation system, which is strongly dependable on the sequence of transcript mRNA. In vitro transcription plays an important role in generating mRNA molecules that can be translated to obtain protein products of interest. Thus, the choice of genes involved in in vitro transcription and translation is crucial for protein or viral production. Several important parameters are involved, e.g., plasmid choices, protein capping, transcription, and translation machinery, with the choice of DNA-dependent RNA polymerase promoters as the most crucial one. The promoter region should be compatible with the DNA-dependent RNA polymerase of choice as it controls the initiation of transcription. The length of the promoter is gene-specific and varies greatly among genes [84].

Presently, the most commonly used promoters for in vitro transcription are bacteriophage T7 [85] and SP6 [86], in addition to the eukaryotic promoter of the human cytomegalovirus, CMV [87]. It has been reported that the incorporation of the T7 and SP6 promoter regions in the construct resulted in the infectious clones of the tobacco mosaic virus [88] and Zika virus [89]. In addition, the CMV promoter has been used in the development of an infectious human norovirus clone in vitro by the Katayama research group and Oliveira et al. [10,11]. However, only the latter group retained viral clone particles due to no additional nucleotide presence in the region. Another important property required in the translation system is the splicing machinery for structured proteins. The most commonly used is hepatitis delta virus (HDV) ribozyme [11,81,89] due to its characteristic of efficient self-cleaved during viral RNA replication.

## 10. Challenges in Cloning of Infectious HuNoV-VLPs

Infectious DNA clones have been used to investigate important virus properties, such as viral infectivity, replication, host specificity, and functions of coding and noncoding genomic regions. On the contrary, cloning many subgenomic cDNA fragments is frequently required for their construction. Despite recent advancements made possible by novel molecular techniques, the process is still time-consuming, and many challenges remain, such as viral sequence instability and bacterial host toxicity [60].

In the case of the human norovirus, due to a lack of a suitable tissue culture system, murine norovirus was used to study the relationship between the basic norovirus replication mechanism in tissue culture and the pathogenesis in a natural host [90], and in vitro transcription and translation [91] have been used as an alternative to developing infectious clones for its molecular mechanism study. The strategy in designing a full-length cDNA in a plasmid backbone is a crucial step to rescuing infectious viral clones. Katayama and colleagues have demonstrated that the construction of full-length cDNA human norovirus with the vTF7 capping enzyme can express nonstructural protein from ORF1 when transfected into human 293t cells but not the structural protein in ORF2 and ORF3. However, a construct that consisted only of ORF2 and ORF3 (subgenomic region) was translated and expressed when transfected into the cells. The study suggested that a cap or a cap-like structure attached to the 5′ end of the genome and subgenome might be required to initiate translation [91].

Furthermore, an infectious viral clone progeny was recovered by Katayama et al. after changing their strategy by incorporating a mammalian elongation factor-1α (EF-1α) promoter into an expression cassette of the HuNoV GII.3 U201 clone genome following unsuccessful attempts to obtain HuNoV genome expression using the CMV promoter. The plasmid vector fused with complete genetic materials for in vitro transcription and translation was transfected into mammalian cells, COS7, which subsequently expressed nonstructural and structural proteins, in addition to genomic- and subgenomic-sized RNAs. The infectious viral clone progeny was analyzed by observation of the GFP signal expressed in the transfected mammalian cells [91].

In comparison, although cloning full-length viral clones into the plasmid vector should be straightforward, it was not the case. Adopting the HuNoV infectious clone construction was a challenge when conventional cloning strategies were used. Hence, via a new technology widely used in the reverse genetics of RNA viruses, the Gibson Assembly, Oliveira et al. successfully constructed the infectious clone of the HuNoV GII.4 Sydney subtype. Carefully designed, the expression cassette incorporates the whole viral genome without adding or deleting nucleotides, and the expression was controlled by the CMV promoter. Difficulties in amplifying the entire viral genome arise when it has to be divided into two fragments with overlapping regions. In addition, low-yield plasmid vector backbone recovery was overcome by reducing the contamination of the plasmid template after the DNA fragment preparation or by reducing the circular form of the plasmid template and size [11]. Hence, a specific and critically structured recombinant DNA must be considered in developing the viral infectious clone construct. The major challenge in developing an infectious clone of HuNoV is finding a suitable culture system to investigate the likelihood function of infectious clone products. The lack of a culture system hindered the investigation of a viral mechanism that mimics natural biological infection.

## 11. Conclusions

Rapid advances in DNA synthesis technology have aided researchers in better understanding the molecular mechanisms of virus–host cell interactions. Despite challenges in the in vitro propagation of certain viruses, particularly the HuNoV, the combination of suitable strategies in the cloning systems and advances in molecular techniques provide feasible initiatives toward producing suitable and efficient infectious VLPs. Nevertheless, crucial parameters, including cDNA synthesis (either from a wild-type virus or a synthetically produced one), construction of a plasmid vector, and cloning strategies require careful consideration, responsible for a viable viral clone recovery, which subsequently influences virulence or attenuation of the resulting transcript. With regard to the HuNoV, the recent cloning system of a seamless assembly and the use of a cell-free system presented the most plausible approaches to obtaining an infectious viral clone.

## Figures and Tables

**Table 1:** The HuNoV-VLP expression systems.

Expression Vector	Assembly Method	Promoter	Expression System	Finding	Ref.
***E. coli***	Capsid protein gene of NoV GII.4 cloned into prokaryotic expression vector; pCold III and pCold IV.	Cold-shock protein A (csp A)	Cold-shock expression system	Nov-VLP self-assembly morphologically identical to native virions obtained and exhibits similar binding pattern with tested VLPs assembled in other cells.	[52]
**Recombinant baculovirus vector**	Subcloning of Norwalk virus subgenomic cDNA into plasmid vector	SP6 promoter	Cell-free systems	Self-assembly of capsid protein that resembles native Norwalk virus characteristics.	[48]
**Recombinant vesicular stomatitis virus (VSV) plasmid vector**	Restriction enzyme cloning by digestion of plasmid and inserts with SmaI and XhoI	P10 promoter	Baculovirus expression system	VSV-expressed capsid protein resulted in the formation of HuNoV-VLPs that resemble native virions—morphology and antigenically.	[53]
**Venezuelan equine encephalitis virus (VEE) replicon plasmid vector**	The NV capsid gene was inserted into polycloning site of the VEE PVR21 plasmid vector using overlapping extension PCR resulted in replicon transcript suitable for transfection in mammalian cells.	Subgenomic 26S promoter	Mammalian system	Transfection resulted in expression of high concentrations of rNV capsid protein that self-assembled into NV VLPs.	[54]
**Recombinant Baculovirus (Bac-to-Bac system)**	Subcloning capsid gene into recombinant transfer vector in Bam HI and Not I sites. The recombinant baculovirus was used to transfect H5 cells for protein production.	Polyhedrin promoter and the p10 promoter	Baculovirus expression in insect cells (H5)	High yield of expressed Norwalk-like virus capsid protein was obtained.	[55]
**pcDNA3.1(+) vector** [10]	The whole genome of GII.4 HuNoV cDNA was cloned into the vector HindIII–BamHI sites.	Not stated	Mammalian system	The presence of HuNoV VP1 protein detected with expected size enabling further gene study of NTPase.	[56]
**Bacmid**	The VLP genes were cloned into an expression vector as described in [57,58]	Not stated	Expression in insect and mammalian cells	Different expression system used for a consensus sequence of prevalent GII.4 variants showed structurally similar VLPs with mixture of VP1 T = 1, T = 3, and predominantly T = 4 icosehedral symmetry.	[59]

**Table 2:** Methods used for construction of VLP clones.

Cloning Methods		Directional Cloning	Example of Applications	Ref.
**Restriction enzyme cloning**	Two types of restriction enzyme that cut the specific nucleotide sequences in two ways: blunt-end and sticky-end cutters. Then, DNA ligases are used to join or ligate together different strands of DNA [65].	Yes and no	Infectious clone of poliovirus, Coxsackievirus, infectious Japanese encephalitis virus clone, infectious cDNA clone of SARS-CoV-2, and recovery of West Nile virus particles from infectious plasmid.	[67,68,69,70,71]
**TA cloning**	The PCR product with 3′ A overhang cloned directly into a linearized cloning vector with a 3′T overhangs, which tailed with dideoxythymidine triphosphate (ddTTP) using terminal transferase; the fragments are joined by the formation of a phosphodiester bond between the vector’s 5′-phosphate at the 3′-overhanging T residue and the PCR product’s 3′-hydroxyl (OH) group from the overhanging A [66].	No	Used in gene characterization, protein structure, and gene function study by sequencing.	[72,73,74]
**Gibson Assembly (** **Isothermal assembly method)**	Cloning steps involve exposing the overlapping region by digestion of 5′ exonuclease, then annealing of complementary overlaps, later extension and ligation of DNA molecule by Phusion DNA polymerase as well as Taq DNA ligase, respectively. After optimization, the Phusion DNA polymerase was selected for its characteristic of proofreading activity for removing noncomplementary sequences [75,76].	Yes	Lettuce mosaic virus (LMV); reproduction efficiency of human adenovirus (HAdV) infectious clone was similar to wild-type strain; similar characteristic foci of rescued dengue virus (DENGV) infectious clone with original virus stock; and a successful construction of full-length genomic clone of human norovirus.	[11,77,78,79]

## Data Availability

Not applicable.
